# Testing the validity of a self-report scale, author recognition test, and book counting as measures of lifetime exposure to print fiction

**DOI:** 10.3758/s13428-021-01784-2

**Published:** 2022-03-11

**Authors:** Lena Wimmer, Heather J Ferguson

**Affiliations:** 1 Department of Education, University of Freiburg, Rempartstr., 11, 79098 Freiburg im Breisgau, Germany; 2 School of Psychology, University of Kent, Canterbury, UK

**Keywords:** Fiction, Print exposure, Reading habits, Author recognition test, Home literacy

## Abstract

We report a study testing the validity of the three most commonly used indicators of lifetime exposure to print fiction, namely a self-report scale, an author recognition test (ART), and book counting, in a sample of older adults (*N*=306; *M*_age_ = 59.29 years, *SD*_age_ = 7.01). Convergent validity of the self-report scale and book counting was assessed through correlations with the fiction sub-score of the ART; divergent validity of these two indicators was examined via correlations with the non-fiction sub-score of that ART. We also assessed criterion-related validity by testing the degree to which each of the three indicators predicted participants’ performance in a vocabulary test. The self-report scale and book counting were significantly more positively associated with the ART fiction sub-score than the ART non-fiction sub-score. Regression analyses, controlling for gender and non-fiction exposure, revealed that the ART fiction sub-score had the highest explanatory power among all indicators under investigation for predicting vocabulary test performance. The present results suggest that only ARTs may have satisfactory levels of both construct and criterion-related validity. Recommendations for the assessment of fiction exposure and future directions are discussed.

## Introduction

Over recent years, there has been increasing scientific interest in understanding the potential benefits of reading fiction for a range of psychological outcomes. Whilst the majority of empirical research has tested the idea that reading fiction promotes social cognitive abilities including theory of mind, empathy (e.g. Bal & Veltkamp, 2013; Djikic et al., 2013b; Kidd & Castano, 2013), and related outcomes including moral cognition (e.g. Johnson et al., 2013; Koopman, 2015), benefits of reading fiction have been reported in other outcomes as well, for instance the need for cognitive closure (Djikic et al., 2013a), creativity (Black & Barnes, 2021), or changes in personality (Djikic et al., 2009). Across outcome variables, the majority of experimental studies have investigated the effects of reading short fictional narratives. A meta-analysis concluded that such experiments on average yield small-sized benefits for social cognition (Dodell-Feder & Tamir, 2018).

However, recently proposed models by Consoli (2018) and Mar (2018) that outline conditions under which reading (fictional) narratives can lead to psychological benefits question the validity of these findings. According to these models, effects in the sense of deep learning are predicted exclusively after frequent exposure to fictional stories over a prolonged period of time, and not after a single brief reading assignment. One method of testing this prediction is to investigate the effects of lifetime exposure to print fiction: Instead of testing outcomes after a reading task, researchers could assess the amount of fiction participants have read in their lifetime so far, and examine whether the amount of lifetime fiction reading is associated with purported benefits. This research agenda requires valid indicators of lifetime fiction reading. In this article, we report a study investigating the validity of three such indicators.

According to a meta-analysis by Mol and Bus (2011), the most frequently applied indicators of print exposure are self-report scales, book counting, and author recognition tests. Using self-report scales, participants report on their own reading preferences and/or habits, often by responding to items using a rating scale. Prior to publication of the Self-Report Habit Index for Reading (SRHI-R; Schmidt & Retelsdorf, 2016), empirically validated self-report instruments did not exist so that researchers relied on bespoke scales (for examples see Acheson et al., 2008; Spear-Swerling et al., 2010). Schmidt and Retelsdorf (2016) reported some evidence suggesting criterion-related validity of the SRHI-R, as this questionnaire was a stronger predictor of reading achievement and decoding speed than self-reported reading frequency. However, the SRHI-R was no longer a significant predictor of reading achievement when intrinsic reading motivation was included as predictor. Furthermore, the SRHI-R assesses general reading habits, and not fiction reading specifically. Hence, in the present study we used a bespoke single-item self-report scale. We decided to use a single item since the application of a multi-item instrument would have required additional piloting to clarify the instrument’s factor structure.

Book counting, understood as the number of fiction (or non-fiction) books in one’s home—in other words, the size of one’s home library—is typically used within large sociological surveys as an indicator of the home literacy environment. Within Bourdieu's (1984) classic *cultural reproduction theory*, home libraries are regarded as a component of a family’s cultural capital. Elite families are thought to provide their children with high-status cultural signals, including a large home library, in order to convince teachers of their children’s academic excellence. This is supposed to motivate the teachers to give these children extra pedagogical support and seal educational benefits in the long run. More contemporary views, such as the *scholarly culture theory* (Evans et al., 2010), assume that raising children in “bookish” environments, including large home libraries, provides the foundation of their trait-level tastes, skills, and knowledge. This is expected to promote future educational and occupational achievements. In sum, both cultural reproduction theory and scholarly culture theory would predict that the number of books in one’s home is positively linked with reading skills. An important methodological limitation of book counting is that previous studies have not precisely counted participants’ books, but asked them to give rough estimates. For instance, in the study reported by Sikora et al. (2019), participants chose from the following response options: *10 books or less; between 11 and 25 books; between 26 and 100 books; between 101 and 200 books; between 201 and 500; more than 500 books*. To increase precision and attain an indicator of *fiction* exposure, the present study asked participants to count the fiction books in their homes and provide the exact figure.

Author recognition tests (ARTs), first introduced by Stanovich and West (1989), attempt to provide an objective measure of lifetime print exposure. In this task, respondents must identify the real authors from a list of names that includes both real authors and non-authors (so-called foils). The more authors are accurately recognised, the higher the estimated lifetime print exposure. The presumed relation between author recognition and reading amount draws on the assumption that people encode author names for the texts they read. Thus, the more they read, the more author names they should recognise. However, test scores are culturally and temporally sensitive, meaning that recognition of authors varies considerably across countries and short periods of time (McCarron & Kuperman, 2021; Moore & Gordon, 2015). This demonstrates the need to regularly develop updated versions for given cultural contexts. Therefore, in the present study we applied the Author Recognition Test–Genres (ART-G; Mar & Rain, 2015), since it is the most recent version providing separate scores for exposure to fiction and non-fiction.

The above-mentioned meta-analysis by Mol and Bus (2011) provides some evidence on the validity of print exposure measures. Here, 99 studies investigating leisure reading among preschoolers, kindergartners, school children, and higher education students were synthesised. In view of, first, inter-correlations of different types of measures, and second, correlations of these measures with reading skills, it was concluded that print exposure checklists (e.g. ARTs) and book counting have better validity than self-report measures since only the latter are particularly prone to social desirability biases. Despite the importance of the findings it yielded, this meta-analysis was published a decade ago and is limited to child, adolescent, and young adult samples. Hence, we conducted a comprehensive and updated literature search that also considered middle-aged and older adults.

In order to determine criterion-related validity, understood as the degree to which a measure is associated with a behavioural manifestation of the construct to be measured, we chose vocabulary, defined as word knowledge, as criterion of print exposure. This is because word knowledge is regarded as a central component of reading comprehension (e.g. Perfetti & Stafura, 2014), implying that vocabulary should improve as a result of frequent print exposure (Cunningham, 2005). A database search was carried out using PsycINFO and Web of Science, with the search terms “(author recognition test OR print exposure OR home literacy environment OR reading frequency OR leisure time reading) AND (vocabulary OR word knowledge)”. Further studies were identified via reference lists and article recommendations on journal webpages. In total, we detected 117 studies which reported concurrent correlations between at least one print exposure index and vocabulary in participants’ primary language, and which were not included in the meta-analysis by Mol and Bus (2011). Table 1 provides an overview including correlation coefficients with vocabulary; for an extended version also listing inter-correlations of print exposure measures and correlations involving measures of divergent validity, see https://osf.io/ytudn/.

**Table 1:** Overview of studies that provide information on the validity of print exposure measures in terms of concurrent correlations with primary language vocabulary; not included are studies synthesised by Mol and Bus (2011).

Reference	Type of print exposure addressed	Measures of print exposure under investigation	Sample	Concurrent correlation of print exposure measure with vocabulary
Current study	Fiction	ART, book count, self-report	***N*** = 306 older adults	ART: .57 Book count: .31 Self-report: .24
Aram et al. (2013)	General	Parent-report, researcher observations, book count	*N =* 89 children	Home literacy score (composed of all measures listed in the 3rd column): .41
Baroody and Diamond (2012)	General	Parent-report, book count	*N =* 81 preschool children	Home literacy environment score (contains book count, frequency of parental reading to child): .12
Bojczyk et al. (2015)	General	Parent-report, book count	*N =* 112 preschool children from rural or urban region	Urban background: Stipek formal home learning activities (SFHLA) – Peabody Picture Vocabulary Test (PPVT): .17 Stipek informal home learning activities (SIHLA) – PPVT: .09 Home learning experience subscale (HLES) – PPVT: .00 Stony Brook family reading survey (SBFRS; contains book count, frequency of child looking at books) – PPVT: .12 Rural background: SFHLA – PPVT: .44 SIHLA – PPVT: .58 HLES – PPVT: .47 SBFRS – PPVT: .42
Bojczyk et al. (2018)	General	Parent-report, book count	*N =* 112 mother–child dyads participating in Head Start	Stipek Home Learning Activities (SHLA): .38 Home-Learning Environment Profile (HLEP): .24 Stony Brook family reading survey (SBFRS; contains book count, frequency of child looking at books): .27
Bojczyk et al. (2019)	General	Parent-report, book count	*N* = 198 kindergarten children and first-grade pupils	Kindergarten children: Stipek Home Learning Activities (SHLA) – Peabody Picture Vocabulary Test (PPVT): .01 SHLA – Expressive Vocabulary Test (EVT-2): −.01 Home learning experience subscale (HLES) – PPVT: −.03 HLES – EVT-2: .04 Stony Brook family reading survey (SBFRS; contains book count, frequency of child looking at books) – PPVT: .33 SBFRS – EVT-2: .30 First-grade pupils: SHLA – PPVT: −.04 SHLA – EVT-2: −.05 HLES – PPVT: −.16 HLES – EVT-2: −.14 SBFRS – PPVT: .12 SBFRS – EVT-2: .17
Bracken and Fischel (2008)	General	Parent-report, book count	*N* = 233 preschool children from low-income backgrounds	Child reading interest (contains child reading frequency): .23 Parent–child reading interaction (contains number of books and frequency of shared reading): .39
Brittnacher (2015)	General	Parent-report	*N* = 121 preschool children attending Head Start	Enrichment activities (literacy activities the child participated in during a typical week): .10 Dialogic reading (quantity of the parental instructional interactions within literacy activities): .19
Brysbaert et al. (2020), Study 1	Fiction	ART	*N =* 195 undergraduates	Yes/No Vocabulary test Dutch: .05 Vocabulary test Dutch: .42
Brysbaert et al. (2020), Study 3	Fiction	ART	*N =* 85 adults	.28
Brysbaert et al. (2020), Study 4	Fiction	ART	*N =* 72 vocation higher education students	.33
Brysbaert et al. (2020), Study 5	Fiction	ART	*N =* 62 undergraduates and graduates	Yes/No Vocabulary test Dutch: .26 Vocabulary test Dutch: .64
Burris et al. (2019)	General	Title recognition test completed by parents, parent-report, book count	*N =* 256 preschool children attending Head Start	Peabody Picture Vocabulary Test Book count: .22 Frequency of primary caregiver reading to child: −.09 Frequency of other person reading to child: −.06 Frequency of library visits: −.03 Age at which parents began reading to child: .00 Title recognition test: .07 Expressive One Word Picture Vocabulary Test Book count: .14 Frequency of primary caregiver reading to child: .04 Frequency of other person reading to child: −.07 Frequency of library visits: .05 Age at which parents began reading to child: −.03 Title recognition test: .12
Carlson et al. (2012)	General	Parent-report	*N* = 3,104 preschool children receiving special education	.14
Carroll et al. (2019)	General	Parent-report	*N* = 55 preschool children	.36
Chen and Fang (2015)	Popular vs high-brow literature	ART, self-report	*N =* 358 college students	Time spent reading print-based: .15Time spent reading internet-based: .01Reading frequency of fiction, non-fiction, Newspapers and magazines: .15Reading frequency of e-fiction, e-news, blogs, and bulletin board systems: .03ART score: .23
Chen and Fang (2016)	General	Self-report, diary, ART, title recognition test	*N =* 318 fifth graders	Diary - school-day book reading time: .22Diary - non-school-day book reading time: .22Diary - average book reading time: .25 Self-reported recreational reading attitude: .35 Self-reported academic reading attitude: .28Self-reported reading attitude total: .34Self-reported activity preference reading: .35Title recognition test: .23ART: .42
Chow et al. (2017)	General	Self-report, book count	*N =* 312 children aged 3 to 11	Reading resources and opportunities at home (contains book count): .31 Time when parental instruction began: .02 Literacy activities: .02 Duration of daily parental instruction: .08 Parents’ literacy teaching methods: .04
Conte et al. (2020)	General	Self-report, book count	*N =* 494 middle and high school pupils	Book count: .61 Self-reported reading habits: .41
Dąbrowska (2018)	General	ART	*N =* 90 adults	.60
Dąbrowska (2019)	General	ART, self-report	*N =* 90 adults	ART: .60 Self-report: .36
De Jong and Leseman (2001)	General	Parent-report	*N =* 69 third graders	Parent-reported opportunity for literacy interactions: .30
Dulay et al. (2018)	General	Parent-report, book count	*N* = 673 3- to 5-year-old children and their families	Receptive vocabulary Home literacy activities: .06 Home literacy resources (contains book count): .11 Expressive vocabulary Home literacy activities: .05 Home literacy resources: .14 Book vocabulary Home literacy activities: .05 Home literacy resources: .13
Duursma et al. (2007)	General	Book count	*N* = 96 fifth-grade Latino English language learners	.50
Emmorey et al. (2016)	General	ART, magazine recognition test	*N =* 28 deaf adults	Correlations controlled for nonverbal IQ ART: .43 Magazine Recognition Test: .61
Ergül et al. (2021)	General	Parent-report	*N =* 441 kindergarten children	Receptive vocabulary Parent-reported home reading: time 1: .23, time 2: .23Parent-reported shared reading: time 1: .18, time 2: .13 Parent-reported phonological and print awareness activities: time 1: .08, time 2: .16 Expressive vocabulary Parent-reported home reading: time 1: .30, time 2: .27Parent-reported shared reading: time 1: .17, time 2: .15 Parent-reported phonological and print awareness activities: time 1: .05, time 2: .09
Esmaeeli et al. (2018)	General	Parent-report, book count	*N =* 1,171 children	Correlations with emergent literacy (contains vocabulary) Access to print (contains book count): .25 Literacy-related activities: .24 Child interest: .36
Esmaeeli et al. (2019)	General	Parent-report, book count	*N =* 208 children	Home literacy environment index (contains frequency of reading to child and book count): .46
Foster et al. (2005)	General	Parent-report, book count	*N =* 325 families	Parent-reported reading to child: .12Parent-reported books and reading materials: .04
Freed et al. (2017)	General	ART, self-report	*N =* 357 young adults	Extended Range Vocabulary ART: .58 Reading Habits: .26 Advanced Vocabulary ART: .46 Reading Habits: .28 Nelson-Denny Vocabulary ART: .58 Reading Habits: .37
Froiland et al. (2014)	General	Parent-report, book count	*N =* 76 children participating in Head Start	Home literacy environment index (involves book count, frequency of shared reading): .24
Georgiou et al. (2021)	General	Parent-report, book count	*N* = 172 children followed from grade 1 to grade 3	Parent-reported direct teaching: .02 Parent-reported shared book reading: .07 Book count: .24
Gonzalez et al. (2017)	General	Parent-report, book count	*N =* 252 mothers and their preschool children	Receptive vocabulary Home literacy environment (contains frequency of shared reading): .11 Frequency of parent reading to child: .19 Book count: .23 Expressive vocabulary Home literacy environment: .08 Frequency of parent reading to child: .11 Book count: .20
Goodrich et al. (2021)	General	Parent-report, book count	*N =* 944 preschool children	Picture book count: −.02 Alphabet book count: −.01 Frequency of reading to child: −.02 Frequency of parents engaging children in stories: .01
Grant et al. (2011)	General	Title recognition test	*N =* 29 grade 3 pupils	.51
Grant (2012, Study 2)	General, fiction, non-fiction	Self-report, Book cover recognition test, title recognition test	Pupils tested in grades 1 (*N* = 42) and 2 (*N* = 40)	Grade 1 (correlations controlled for age) Book cover recognition test (BCRT) – titles: .40 BCRT – characters: .21 BCRT – details: .21 Home reading: percentage of fiction: −.15 Home reading: number of books read: −.36 Grade 2 (correlations controlled for age) Title recognition test (TRT) overall: .33 TRT fiction: .21
Grant (2012, Study 3)	General, fiction, non-fiction	ART, magazine recognition test	*N* = 97 undergraduate students	ART: .32 ART fiction: .34 Magazine recognition test: .28
Griffin and Morrison (1997)	General	Parent-report, book count	*N =* 295 children followed from kindergarten to grade 2	Home literacy environment index (includes number of books and parent-reported frequency of reading to child): .63
Grolig et al. (2017, Study 2)	General	Parent-report, title recognition test (completed by children)	*N =* 202 preschool children	Explaining concepts vocabulary test Title recognition test (TRT): .39 Home literacy environment index (contains book count, frequency of reading to child): .43 Picture naming vocabulary test TRT: .31 Home literacy environment index: .38
Grolig et al. (2019)	General	Parent-report, title recognition test (completed by children)	*N =* 201 preschool children	Explaining concepts vocabulary test Title recognition test (TRT): .43 Home literacy environment index (contains book count, frequency of reading to child): .34 Picture naming vocabulary test TRT: .43 Home literacy environment index: .38
Hindman and Morrison (2012)	General	Parent-report	*N =* 229 preschoolers	Home literacy environment index (contains frequency of literacy teaching): .04 Frequency of shared book reading: .20
Hofslundsengen et al. (2019)	General	Parent-report	*N =* 111 preschoolers	Home literacy environment index (involves weekly duration of shared book reading): .33
Hutton et al. (2020)	General	Parent-report	*N =* 47 3–5-year-olds	Shared reading score: .45
Inoue et al. (2018)	General	Parent-report, book count	*N =* 214 children followed from kindergarten to grade 3	Parental letter teaching: .23 Parental letter sound teaching: .25 Parental reading words teaching: .15 Parent-reported reading to child: .31 Book count: .19
Iruka et al. (2018)	General	Parent-report, book count	*N =* 5,046 toddlers	Home literacy environment (involves book count, parent–child literacy activities): .24 Frequency of reading to/with child: .08
James et al. (2018)	General	ART, self-report	*N =* 117 adults	Reading time estimate survey: .10Comparative reading habits survey: .35 ART: .45
Johns et al. (2018)	General	Magazine recognition test	*N =* 35 young adults	.55
Johnson et al. (2008)	General	Parent-report, book count	*N* = 455 kindergarten and grade 1 students	Child is read to: .08 Child owns more than 30 books: .21 Child amuses self with books: −.05 Number of books child brings home: .11 Family uses library card more than once a year: .17 Number of household subscriptions: .32
Kalia and Reese (2009)	General	Parent-report	*N =* 50 kindergarten children	Book-reading practices: .34 Teaching practices: .29
Kim et al. (2018)	General	ART, magazine recognition test	*N =* 60 undergraduate students	Print exposure index (composed of ART and magazine recognition test): .38
Korat et al. (2013)	General	Parent-report, book count	*N =* 109 kindergarten children	Home literacy environment index (contains book count, frequency of reading to child) Spoken vocabulary: .38 Written vocabulary: .46 Peabody Picture Vocabulary Test: .18
Landi (2010)	General	ART	*N =* 928 university students	.46
Lee et al. (1997)	General	ART, self-report	*N =* 30 adults	ART: .53 Frequency of reading for pleasure: .59
Lee et al. (2019)	General	ART, self-report	*N =* 104 undergraduates and graduates	Self-reported reading time: .04 Comparative reading habits survey: .23 ART: .35
Lehrl et al. (2020)	General	Parent- and self-report, researcher observations, book count	*N* = 554 children followed from age 3 to age 13	Formal literacy activities score (contains frequency of literacy teaching activities): −.02 Informal literacy activities (contains frequency of reading to child & book count): .41
Lenhart et al. (2021)	General	Parent-report	*N =* 643 children	Expressive vocabulary: .17 Receptive vocabulary: .17
Leseman and De Jong (1998)	General	Parent-report	*N =* 89 primary school children	Parent-reported frequency of literacy-related activities with child Age 4: .46 Age 7: .30
Lewis et al. (2016)	General	Parent-report, book count	*N =* 93 preschool children attending Head Start	Book count: −.05 Frequency of shared reading: .00 Frequency of maternal teaching: −.24
Li et al. (2008)	General	Parent-report, book count	*N* = 88 children followed for 3 years starting at age 5	Beijing sample Home reading resources (contains book count): .12 Exposure to reading at home: .09 Direct literacy teaching at home: .40 Exposure to reading in classroom: .35 Direct literacy teaching in classroom: .42 Hong Kong sample Home reading resources (contains book count): .14 Exposure to reading at home: .34 Direct literacy teaching at home: .56 Exposure to reading in classroom: .72 Direct literacy teaching in classroom: .60
Li et al. (2020)	General	Parent-report, book count	*N =* 124 pupils in grades 4 to 6	Book count: .59 Age at which child started shared reading: .43
Kara et al. (2014)	General	Parent-report, book count	*N* = 500 children aged 8–36 months	Number of child books: .02 Number of adult books: .05 Parent–child interactions (contains teaching letters/words, shared reading): .16
Liu et al. (2018)	General	Parental self-report, book count	*N =* 140 kindergarten children	Parent-reported frequency of literacy teaching: .04 Parent-reported frequency of story reading to child: .13 Book count: .35
Manolitsis et al. (2011)	General	Parent-report, book count	*N* = 70 children followed from kindergarten to grade 4	Parent-reported frequency of parental literacy teaching: −.05Storybook exposure (contains number of books and parent-reported frequency of reading to child): .38
Manolitsis et al. (2013)	General	Parent-report, book count	*N =* 82 children followed from kindergarten to grade 1 and their parents	Parent-reported frequency of parental literacy teaching: .19Storybook exposure (contains book count and parent-reported frequency of reading to child): .33
Mar and Rain (2015), Study 1	Fiction and non-fiction	ART, self-report	*N =* 349 undergraduates	Self-reported fiction exposure: .23 ART fiction: .32
Marjanovič-Umek et al. (2008)	General	Parent-report, title recognition test completed by parents	*N =* 115 preschool children	Correlations with Language Expression Scale (contains vocabulary) Home literacy environment score: .15 Title recognition test: 15
Marjanovič-Umek et al. (2017)	General	Parent-report	*N =* 51 toddlers	Frequency of shared reading: .34
Martin et al. (2009)	General	ART	*N* = 646 twin pairs and 307 singleton siblings of twins in the adolescent to young adult age range	.42
Mendez (2010)	General	Parent-report	*N =* 288 children attending Head Start	Frequency of reading to child: .14
Meng (2015)	General	Parent-report	*N* = 2,611 preschool children attending Head Start	Home literacy environment index (reflects parental teaching and reading behaviours, providing literacy activities, making literacy materials accessible for child): .10
Mesman and Kibby (2011)	General	Title recognition test	*N =* 158 8- to 12-year-olds	.33
Misyak and Christiansen (2012)	General	ART	*N =* 30 undergraduate students	.33
Moore and Gordon (2015)	General	ART	*N* = 1,012 university students	.44
Napoli and Purpura (2018)	General	Parent-report	*N =* 114 preschool children	Home literacy environment – code-related (contains frequency of printing letters, identifying letters, and identifying letter sounds): .30 Frequency of storybook reading: .01
Niklas and Schneider (2013)	General	Parent-report, book count	*N =* 921 children followed from kindergarten to grade 1	Home literacy environment index (contains parent-reported child reading frequency, parent-reported frequency of reading to child, book count): .60
Niklas et al. (2013)	General	Parent-report, book count	*N =* 922 kindergarten children	Cultural capital (contains book count): .55 Cultural praxis (contains frequency of reading to child, library visits): .46
Niklas and Schneider (2015)	General	Parent-report, book count	*N* = 125 preschool children	Home literacy environment index (contains frequency of reading to child, book count): .20
O’Brien et al. (2020)	General	Parent-report, book count	*N =* 1,327 kindergarten children and their parents	Shared reading score (includes book count, parent-reported frequency of shared reading): .20 Parent habit score (contains frequency of parental literacy teaching activities): .09 Child interest score (contains frequency of child looking at books, asking to be read to): .07
Ocal (2016)	General	ART	*N =* 42 university students	.43
Patterson (2002)	General	Parent-report	*N* = 64 bilingual 21- to 27-month-old children from homes in which Spanish and English were spoken	Frequency of reading to child in English – English vocabulary: .40 Frequency of reading to child in Spanish – Spanish vocabulary: .35
Payne et al. (2012)	General	ART, self-report	*N =* 139 older adults (taken from the Senior Odyssey project)	ART: .62
Peeters, Verhoeven, de Moor, et al. (2009a)	General	Parent-report, book count	*N =* 35 children with cerebral palsy	Child writing experiences: .12 Child experiences of literacy materials: .14 Child storybook reading: .07 Child story orientation activities: .31 Child word orientation activities: .08 Provision of literacy materials (contains book count): −.12
Peeters, Verhoeven, van Balkom, and de Moor (2009b)	General	Parent-report, book count	*N =* 40 6-year-olds with cerebral palsy, *N* = 62 age-matched typically developing controls	Children with cerebral palsy Child writing experiences: .29 Child experiences with literacy materials: .23 Child storybook reading interest: .04 Child story orientation activities: .19 Child word orientation activities: .26 Child book orientation activities: .04 Provision of literacy materials (contains book count): .04 Typically developing controls Child writing experiences: −.19 Child experiences with literacy materials: .01 Child storybook reading interest: .03 Child story orientation activities: −.17 Child word orientation activities: −.03 Child book orientation activities: −.01 Provision of literacy materials (contains book count): .01
Petrill et al. (2014)	General	Parent-report	*N* = 212 kindergarten children at risk for language impairment	Frequency of storybook reading: .11
Pfost et al. (2013)	Fiction, non-fiction	Self-report	*N =* 1,226 secondary school pupils	Frequency of reading novels, stories, or tales: .34
Pratheeba and Krashen (2013)	General	Self-report	*N =* 25 engineering students	Self-reported reading habits: .78
Prevoo et al. (2014)	General	Parent-report, book count	*N* = 111 6-year-old children of first- and second-generation Turkish immigrant parents in the Netherlands	Dutch vocabulary Frequency of reading by mother: .10 Frequency of reading by father: .23 Book count: .29 Turkish vocabulary Frequency of reading by mother: .13 Frequency of reading by father: .05 Book count: −.03
Reynolds and Werfel (2020)	General	Parent-report	*N =* 22 3- to 4-year-old children with hearing loss and *N =* 27 age-matched controls	Children with hearing loss Parent facilitation of literacy: −.26 Child orientation to literacy: .11 Child interaction with books: .54 Controls Parent facilitation of literacy: .04 Child orientation to literacy: .03 Child interaction with books: −.00
Rose et al. (2018)	General	Observation of parent–child interactions, parent-report, book count	*N =* 547 3-year-olds	Home literacy environment index (contains frequency of shared reading and book count) – language skills (contains vocabulary): .42
Scheele et al. (2012)	Personal narrative, impersonal narrative	Parent-report	*N =* 58 3-year-olds	Impersonal narrative input: .21 Personal narrative input: .47
Schmidtke et al. (2018)	General	ART, magazine recognition test	*N* = 138 adolescents/young adults	Print exposure score (composed of ART and magazine recognition test): .70
Schmitt et al. (2011, Study 1)	General	Title recognition test (completed by parents), parent-report	*N =* 50 infants	MacArthur–Bates Communicative Development Inventory - Words and Gestures Title recognition test: .02 Home literacy environment index (contains frequency of reading to child): .55 Computerised comprehension task Title recognition test: .17 Home literacy environment index: .35
Schroeder et al. (2016)	General	Title recognition test, book count	*N =* 416 primary school children	Title recognition test: .58 Book count: .10
Segers et al. (2016)	General	Parent-report, book count	*N =* 101 kindergarten children	Reading climate (involves book count): .35 Reading frequency (frequency of reading to child, frequency of child asking to be read to): .22
Sénéchal et al. (2018)	General	Self-report	*N =* 103 grade 4 children	Frequency of reading for pleasure: .07
Sénéchal and LeFevre (2014)	General	Parent-report	*N* = 110 children followed from kindergarten to grade 2	Kindergarten Teach/expect (involves parent teaching to read words and parents’ expectations about their child’s reading before grade 1): .08 Shared reading: .31 Grade 1 Teach/listen (involves parent teaching to read words and parents’ listening to their child reading): .11 Shared reading: .40 Grade 2 Teach/listen: −.13 Shared reading: .09
Shriver et al. (2020)	General	Parent-report	*N =* 308 infants at time 1, *N* = 179 infants at time 2	Time 1 (age 11–15 months): .21 Time 2 (age 23–37 months): .13
Sparks et al. (2014)	General	ART, Magazine recognition test	*N =* 54 grade 10 pupils	Print exposure composite score (composed of ART and magazine recognition test) – ISTEP reading (contains vocabulary): .61
Sparks and Reese (2013)	General	Parent-report	*N =* 60 preschool children attending Head Start	Correlations controlled for child age and maternal education Peabody Picture Vocabulary Test Frequency of reading to child: −.04 Frequency of print teaching: −.08 Frequency of reading teaching: −.05 Frequency of child asking to be read to: .10 Expressive Vocabulary Test Frequency of reading to child: .16 Frequency of print teaching: −.06 Frequency of reading teaching: −.08 Frequency of child asking to be read to: .09
Spear-Swerling et al. (2010)	Fiction and non-fiction	ART, self-report	*N* = 87 sixth graders	ART: .57 Reading habits fiction books: .25
Strasser et al. (2017)	General	Book cover recognition test	*N* = 281 first-grade children	.26
Stutz et al. (2016)	General	Self-report	*N =* 1,075 primary school pupils	Self-reported reading frequency – word comprehension girls: .27Self-reported reading frequency – word comprehension boys: .29
Suggate et al. (2011)	General	Parent-report	*N =* 103 primary school children	Home literacy environment index: .23
Tabullo and Gago-Galvagno (2021)	General	Parent-report, book count	*N =* 136 infants	Frequency of shared reading: .17 Book count: .07
Torppa et al. (2007)	General	Parent-report, book count	*N =* 186 preschool children	Children at risk of dyslexia Frequency of shared reading: .37 Access to print (contains book count): .34 Child interest in print: .44 Controls Frequency of shared reading: .25 Access to print: .13 Child interest in print: .12
Torppa et al. (2013)	General	Parent-report	*N* = 1,006 kindergarten children	Frequency of shared reading: .15 Frequency of literacy teaching: −.05
Tremblay et al. (2020)	General	Self- and parent-report, retrospective title recognition test, ART	*N =* 45 adolescent-parent dyads	Self-reported reading enjoyment and frequency: .08 ART: .44
van der Schuit et al. (2009)	General	Parent-report, book count	*N =* 48 children with intellectual disabilities	Receptive language (contains receptive vocabulary) Child’s storybook reading interest: .03 Child’s activities with literacy materials: .25 Provision of literacy materials (contains books count): .06 Story orientation activities: .60 Book orientation activities: .06 Word orientation activities: .40 Productive vocabulary Child’s storybook reading interest: −.01 Child’s activities with literacy materials: .34 Provision of literacy materials: .13 Story orientation activities: .52 Book orientation activities: −.14 Word orientation activities: .33
Vasilyeva et al. (2018)	General	Parent-report, book count	*N =* 1,332 first graders and their parents	Correlations with child literacy score (contains vocabulary) Language activities at home (contains shared book reading): .27 Book count: .20
Veldre et al. (2021)	General	ART	*N* = 49 older adults	.51
Welcome and Trammel (2017)	General	ART, self-report	*N =* 48 adults	ART: .53 Adult Reading History Questionnaire: .10
West et al. (1993)	General	Range of exposure checklists containing author names, magazine titles, newspaper titles; actual leisure reading in a waiting lounge	*N* = 217 adults	Actual leisure reading in a waiting lounge: .46 ART: .62 Magazine recognition test: .48 Newspaper recognition rest: .47
Westerveld et al. (2017)	General	Parent-report	*N =* 57 preschoolers with autism spectrum disorder	Home literacy environment index (contains frequency of shared reading, child asking to be read to): .06
Willard et al. (2015)	General	Parent-report, book count	*N* = 119 preschoolers and *N* = 121 4th graders with a Turkish background living in Germany	Preschool children Home literacy environment index (contains book count, frequency of reading to child): .19 4th graders Home literacy environment index: .09
Yuet-Han Lau and McBride-Chang (2005)	General	Parent-report, self-report, book count	*N =* 92 2nd graders	Correlations controlled for age Self-report Interest: literacy skills learning: −.02 Interest: literacy skills self-efficacy: .17 Interest: bookstore and storybook: .27 Book count: .41 Parent-report Frequency of buying books: .17 Frequency of reading: .16 Duration of reading: −.04 Duration of teaching to read: .10
Zhang et al. (2018)	General	Parent-report, book count (provided by parents), title recognition test (provided by parents), book title knowledge (provided by children)	*N =* 147 kindergarten children	Frequency of shared book reading: .19 Number of children’s books at home/children’s title recognition list: .24 Children’s knowledge of book titles: .28
Zhang et al. (2020)	General	Parent-report, book count	*N =* 145 children followed from kindergarten to grade 2	Reading to child at bedtime: .17 Reading to child at other times: .14 Parents’ diary: .19 Teaching to read characters: .02 Teaching to read pinyin: .02 Book count: .11 Visits of libraries/bookstores: .18
Zucker et al. (2013)	General	Teacher logs	*N =* 178 preschoolers	Fall assessments Frequency of shared reading: −.19 Spring assessments Frequency of shared reading: .01

The vast majority of investigations (82 out of 117 studies in Table 1) have studied child samples up to primary school age, whereas studies with adolescents of secondary school age (8 out of 117 studies in Table 1) and young or middle-aged adults (25 out of 117 studies in Table 1) are less frequent. In addition, there is an apparent lack of studies with older adults (2 out of 117 studies in Table 1: Payne et al., 2012; Veldre et al., 2021). Investigating this population is especially informative for recent models on the effects of reading (fictional) narratives by Consoli (2018) and Mar (2018), since the amount of fiction read accumulates over the lifespan, so that effects of fiction exposure should be largest in older adults compared to younger samples.

Regarding criterion-related validity, the following correlation coefficients between print exposure measures and vocabulary have been observed (see Table 1): for ARTs, 34 correlation coefficients range between .05 (yes/no vocabulary test in Brysbaert et al., 2020, Study 1) and .70 (print exposure index composed of ART and magazine recognition test in Schmidtke et al., 2018), with an interquartile range of .34 to .58. For book count, 71 correlation coefficients range between −.12 (Peeters, Verhoeven, de Moor, et al., 2009a) and .63 (home literacy environment index including book count and parent-reported frequency of reading to child in Griffin & Morrison, 1997), with an interquartile range of .12 to .38. For self-report measures (or parent-report measures in case of younger child samples), 257 correlation coefficients range between −.36 (correlation controlled for age in Grant, 2012, Study 2) and .78 (Pratheeba & Krashen, 2013), with an interquartile range of .06 to .31. Taken together, the pattern of correlations confirms the earlier conclusions by Mol and Bus (2011) that ARTs and book counting have better validity than self-report indicators. Beyond this, the current literature review seems to suggest that ARTs have even better criterion-related validity than book counting.

However, the majority of extant work has relied on a combination of self-report scales with either author recognition tests or book counting, whereas interrelations between all three indicators have rarely been tested so far; a look at Table 1 reveals that only four out of 117 studies included all types of indicators (namely Burris et al., 2019; Grolig et al., 2017, 2019; and Zhang et al., 2018), and that all of these studies worked with child samples. Also, these studies applied title recognition tests instead of ARTs, so do not address ARTs directly. For those studies applying more than one type of index, the following inter-correlations were reported (see https://osf.io/ytudn/): 15 correlation coefficients addressing the association between ARTs and self-/parent-report measures range between .03 (time spent reading online in Chen & Fang, 2015) and .50 (frequency of reading for pleasure in Lee et al., 1997), with an interquartile range of .16 to .41; and 55 correlation coefficients concerning the relation between self-/parent-report measures and book count range between −.02 (Torppa et al., 2007) and .73 (score containing book count and parent-reported frequency of shared reading in O’Brien et al., 2020), with an interquartile range of .20 to .46. There were no studies reporting the association of book count with ARTs. It would therefore be important to assess construct validity, i.e. whether the three types of indicators measure the same or different constructs, more extensively.

A previously understudied question is whether print exposure measures have divergent validity, which would indicate that they do not correlate highly with measures of theoretically unrelated constructs (Campbell & Fiske, 1959). In fact, assessment of divergent validity should be an integral part of each validation process (see also Hodson, 2021): strictly speaking, assumptions about a measure’s convergent validity draw on a comparison of associations with indicators reflecting similar constructs on the one hand and associations with indicators reflecting dissimilar constructs on the other. A measure is said to have good convergent validity if the former associations are considerably higher than the latter. Nevertheless, within the 117 studies listed in Table 1, we identified only 68 correlation coefficients between vocabulary on the one hand and constructs thought to be associated with vocabulary to a lower extent than print exposure on the other (see https://osf.io/ytudn/). These divergent measures reflected various behaviours and skills, including non-verbal intelligence, numeracy, and memory. Correlation coefficients ranged between −.28 (rapid automatised naming test in Zhang et al., 2020) and .61 (inference-making ability in Sénéchal et al., 2018; IQ in Sparks et al., 2014), with an interquartile range of .10 to .31. This seems to suggest that only ARTs may have divergent validity, since ARTs typically correlated more strongly with vocabulary than the divergent measures included in Table 1 (see above), whereas book counting and self-/parent-report measures did not typically exceed correlations of .31. Yet, this assumption may be premature due to the relative scarcity and heterogeneity of investigations into divergent validity. More research in this area would be desirable.

Finally, the field has focused on the amount of lifetime reading in general, but not reading fiction specifically: Only 10 out of the 117 studies reported in Table 1 looked at fiction exposure (namely Brysbaert et al., 2020, Studies 1, 3, 4, 5; Chen & Fang, 2015; Grant, 2012, Studies 2, 3; Mar & Rain, 2015; Pfost et al., 2013; Spear-Swerling et al., 2010). Hence, conclusions about assessment of fiction exposure are currently not supported.

In the present article we report a study investigating the three main indicators of lifetime exposure to written fiction in a sample of older adults (here defined as individuals between 50 and 80 years of age). We examined the construct validity of a self-report scale and book counting, two types of measures whose validity has been evidenced to a relatively low degree, especially regarding the fiction exposure of older adults, against the fiction sub-score of an ART, for which validity is supported by a comparatively larger evidence base. Convergent construct validity was investigated in terms of bivariate correlations of the ART-G fiction sub-score with the self-report scale and book counting. Divergent validity was tested through bivariate correlations of the ART-G non-fiction sub-score with the self-report scale and book counting. In addition, we examined criterion-related validity insofar as we determined the value of each indicator in predicting performance in a vocabulary test.

We aimed to answer the following research questions:

1. How strongly are self-report scales and counting fiction books correlated with fiction author recognition lists on the one hand and non-fiction author recognition lists on the other?
  More positive correlations with fiction author recognition than with non-fiction author recognition would suggest that self-report scales and counting fiction books measure the same construct as the ART-G fiction sub-score, and hence, the most parsimonious indicator might be sufficient to assess lifetime exposure to print fiction.
2. Which of the three indicators demonstrates the strongest positive association with word knowledge?
  This indicator can be regarded as the one with the best criterion-related validity.

## Methods

This study utilised a correlational design and was authorised by the Research Ethics Committee of the School of Psychology at the University of Kent before study commencement. The sample overlaps with the one reported in Study 2 within Wimmer et al. (2021). Wimmer et al. (2021) investigated relations of the ART-G subscales with empathy, theory of mind, general world knowledge, and imaginative skills. As distinct from the present study, Wimmer et al. (2021) did not include self-report measures of fiction reading or book counting.

### Participants

A total of *N =* 337 participants were recruited via Prolific Academic, the University of the Third Age (https://u3a.org.uk/), and through local social media/web pages. Participants were deemed eligible if they were native English speakers and were between 50 and 80 years of age. Participants were excluded from analyses if they did not report their age (*N =* 5), did not pass an attention check item interspersed within the survey (*N =* 11), or selected more than two mock authors in the ART-G^1^ (*N =* 15). This resulted in a final sample of *N =* 306^2^, 281 of which were recruited from Prolific Academic and 25 from one of the other sources named above. The mean participant age was 59.29 years (*SD*_age_ = 7.01), and 60.5% were female. All respondents gave written informed consent prior to data collection and were compensated with a payment of £10.00, either via bank transfer or a digital shopping voucher. Post hoc power analyses using SPSS 27 showed that the final sample size had a power of 1 − β > .99 to detect a medium-sized correlation of *rho =* .30 in a two-tailed test adopting a significance level of *p* < .05, and a power of 1 − β = .41 to detect a small-sized correlation of *rho* = .10 in the same sort of inference test.

### Assessment measures

#### Lifetime exposure to print

*ART-G* (Mar & Rain, 2015) provided the first indicator of reading habits. Respondents were tasked to accurately recognise author names from a list that included 110 fiction authors and 50 non-fiction authors (targets), as well as 40 non-authors (foils). Fiction and non-fiction sub-scores were calculated from the number of selected authors for each genre; i.e., the fiction sub-score is the sum of correctly identified fiction authors, the non-fiction sub-score is the sum of correctly identified non-fiction authors. As distinct from the scoring procedures of the ART version by Stanovich and West (1989), foils were not subtracted from hits because the ART-G materials do not contain instructions to do so. Since we excluded participants selecting more than two foils, the penalty for foil checking was very strict (see above). Hence, the final sample for analyses had limited variance of ART-G foils, and further control measures did not seem necessary. Split-half reliability (Guttman split-half coefficient; test halves were composed using the odd-even method) was .96 for the fiction sub-score and .86 for the non-fiction sub-score.

*Book counting* served as the second measure of print exposure. Participants were given the following instruction: “How many fiction books do you have at home? Fiction books include novels such as crime novels, romantic novels, science fiction novels, but also short stories, comics/graphic novels, fairy tales, storybooks (often for children), theatre plays, poetry, etc. Please also include fiction e-books you may have on your e-book reader. If you live with other people, please only count the books that belong to you (i.e. reflect YOUR reading preferences). If you have more than 160 fiction books in your house, you can stop counting when you have reached 160 fiction books.” Participants were explicitly asked to give an accurate response and were reminded that failure to do so would make the study results useless. The threshold of 160 books was based on the finding that British households have on average 143 books (*SD =* 179; Sikora et al., 2019), but this score does not differentiate between fiction and non-fiction. Since there is no information available regarding the proportion of fiction versus non-fiction books typically owned, we arbitrarily assumed that, on average, 50% of books at home (*M* = 72, *SD* = 80) are fiction, and 50% are non-fiction (*M* = 72, *SD* = 80). Participants having more than (*M* + 1 *SD* =) 152 fiction books can therefore be considered scoring above average. Assuming that the number of books in one’s home is normally distributed and that the current sample was representative, collecting precise book counts from everyone scoring below 152 implied that we were able to gather precise estimates from approximately 84% of the sample. The number 160 rather than 152 was used as a cut-off to provide participants with a round number, which made instructions easier to follow, and to get exact estimates from even more than 84% of the sample. The use of this threshold was regarded as feasible because it increased feasibility for participants, and prevented inaccurate responses.

Thirdly, participants *self-reported* on their reading frequency by responding to “About how often do you read a fiction book?” using a six-point Likert scale with response options being 1 = “less than once a month”, 2 = “once a month”, 3 = “more than once a month”, 4 = “once a week”, 5 = “more than once a week”, and 6 = “every day”.

#### Vocabulary

An adapted version of the vocabulary subtest of the Wechsler Abbreviated Scale of Intelligence–Second Edition (WASI-II; Wechsler, 2011) reflected the breadth of participants’ vocabulary and overall word comprehension. Respondents had to provide a written definition of 31 words presented to them. The time limit for each word was 30 s. Correct responses were awarded a score of 2, partly correct responses were coded 1, and incorrect responses received a score of 0. A sum score with a possible range of 0 to 62 served as dependent measure. Split-half reliability (Guttman split-half coefficient; test halves were composed using the odd-even method) was .84.

In the original version, the examiner conducts the vocabulary test in a face-to-face session with the participant. The test was adapted to fit the online setting of the study: participants received the same instructions and items, and were also given the same time limit as in the original version. As distinct from the original version, instructions were presented in written from via a Qualtrics survey instead of orally by the examiner, and participants typed their responses into a text field instead of answering orally.

### Procedure

Volunteers participated online, via the Qualtrics platform. After providing informed consent, participants completed the vocabulary test, ART-G, book counting survey, and self-report reading scale in that order. Finally, they provided their demographics and were debriefed in written form and remunerated. Participants also completed other tasks reported in Wimmer et al. (2021). The entire study took approximately 90 min per participant.

### Data analysis

Full data and analysis scripts are available on the Open Science Framework web pages (see https://osf.io/ytudn/ for data and https://osf.io/sb7xz/ for analysis scripts). If not otherwise stated, statistics were computed using SPSS 27 software. After computing descriptive statistics of our key variables, we conducted Kolmogorov–Smirnov tests to check whether the measures of print exposure were normally distributed. Bivariate correlations between all print exposure measures and vocabulary were analysed through bivariate Spearman correlations. Next, we compared the correlation coefficients observed for the ART-G fiction sub-score with the ones observed for the ART-G non-fiction sub-score using an online calculator (https://www.psychometrica.de/correlation.html). We also checked whether the three indicators of fiction exposure and the vocabulary sum score were associated with age (using bivariate correlations) or gender (using independent-samples *t*-tests), to see whether analyses would have to be controlled for age and/or gender. Associations of the three indicators of fiction exposure with the vocabulary sum score were tested using a hierarchical linear regression, with the vocabulary sum score serving as the outcome variable. The self-report reading frequency scale was entered as first predictor, followed by the book count, and finally the ART-G fiction sub-score. Unless otherwise mentioned, we adopted the standard 5% significance level.

## Results

**Fig. 1:**
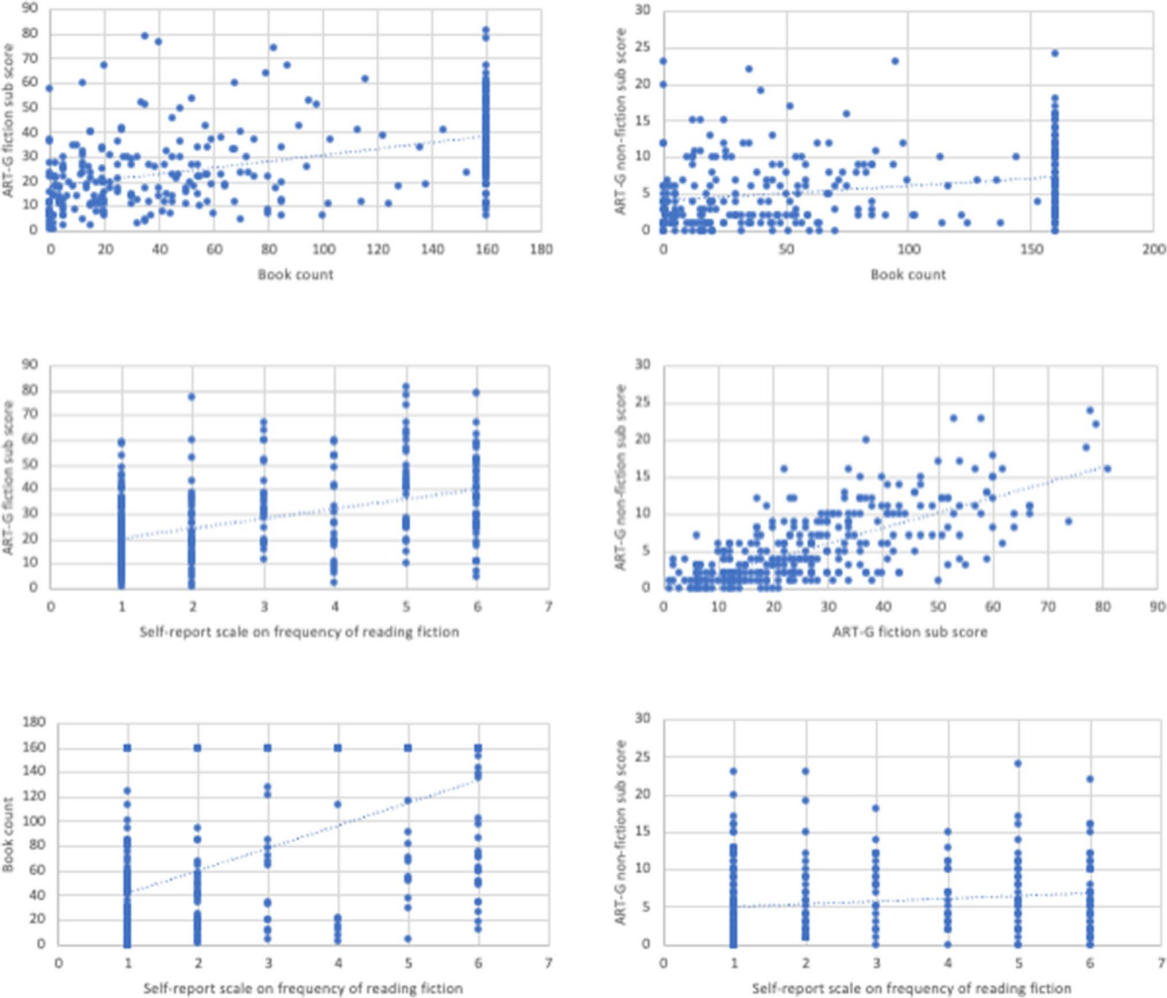
Regression plots illustrating inter-correlations of the indicators of print exposure under investigation

Descriptive statistics for all independent variables and the dependent measure are summarised in Table 2. Significant Kolmogorov–Smirnov tests indicated that none of the indicators of print exposure under investigation was normally distributed (all *p*s < .001). In line with this, for the book count, 30.4% of the current sample reported having 160 or more fiction books in their homes, whereas the expected percentage under normal distribution is 13.5%. Hence, interrelations between the ART-G fiction and non-fiction sub-scores, the book count, and the self-report scale on the frequency of reading fiction, and the vocabulary sum score were tested using bivariate Spearman correlations, as described above (see Fig. 1 for illustrations). The significance level was adjusted for multiple comparisons using the Bonferroni method, resulting in *p*_crit_ = .005. All correlation coefficients were small- to medium-sized and significant (all *p*s < .0004; see Table 1). The ART-G fiction and non-fiction sub-scores were strongly positively correlated. Nevertheless, the ART-G fiction sub-score was significantly more positively correlated with the book count and the self-report scale than the ART-G non-fiction sub-score (*Z*s > 4.70).

**Table 2:** Descriptive statistics and bivariate correlations for indicators of print exposure and vocabulary

Variable	Descriptive statistics		Spearman’s *rho* correlation coefficients			
	*N*	*M (SD)*	1	2	3	4
1 ART-G fiction sub-score	306	27.28 (17.06)	-			
2 ART-G non-fiction sub-score	306	5.65 (4.91)	.688***	-		
3 Book count	306	73.83 (63.80)	.512***	.317***	-	
4 Self-report scale on frequency of reading fiction	293	2.72 (1.94)	.440***	.206***	.552***	-
5 Vocabulary sum score	306	47.26 (7.65)	.572***	.449***	.310***	.241***

ART-G = Author Recognition Test–Genres; *** *p* < .001

Further analyses revealed that none of the independent variables or the dependent measure were associated with age (*p*s > .051). The vocabulary sum score did not differ by gender (*p* = .425). However, there were significant gender differences for the self-report scale on frequency of reading fiction, *t*(267.84) = −2.192, *p* = .029, *d =* −0.255, book count, *t*(267.48) = −2.329, *p* = .021, *d =* −0.269, and ART-G fiction sub-score, *t*(294.12) = −2.185, *p* = .030, *d =* −0.243. Females scored consistently higher than males. Means (*SD*s) were 2.92 (2.02) vs 2.43 (1.79) for the self-report scale, 80.56 (64.83) vs 63.53 (61.03) for book count, and 28.91 (18.43) vs 24.79 (14.45) for the ART-G fiction sub-score.

**Fig. 2:**
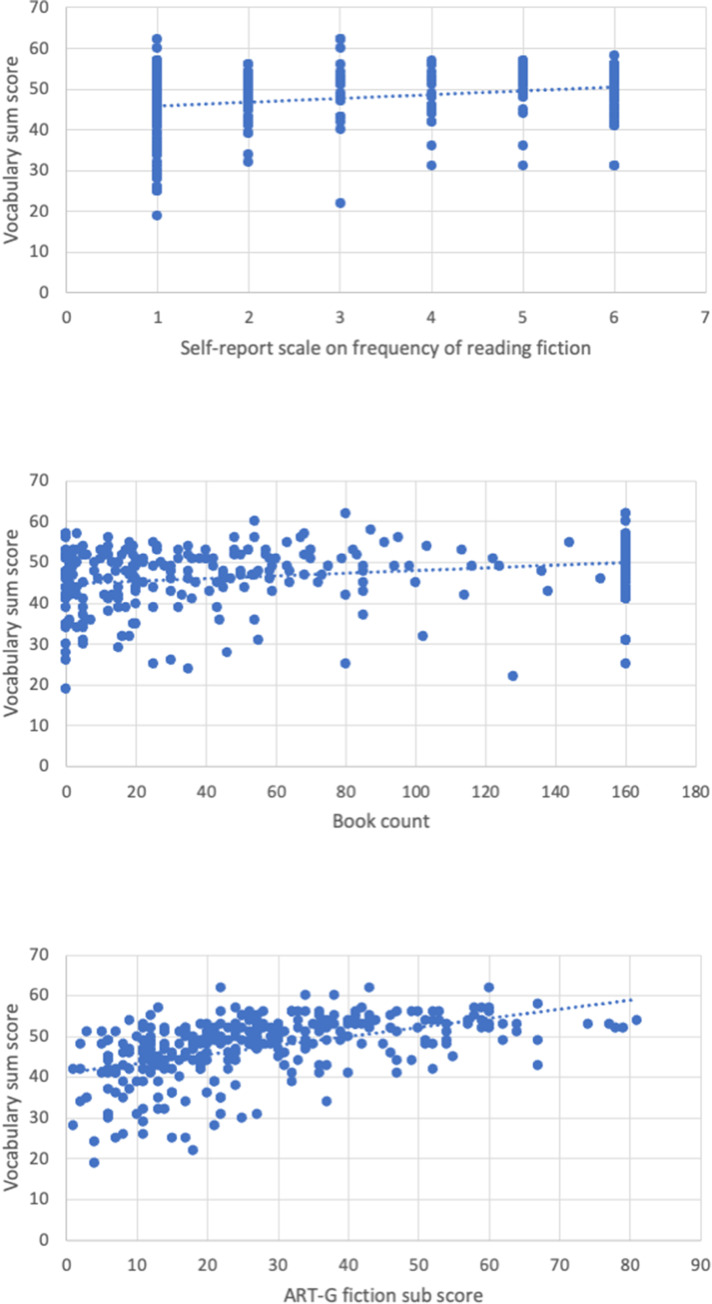
Regression plots illustrating the relationship of the three indicators of fiction exposure with performance in a vocabulary test

As outlined above, a hierarchical regression tested the predictive power of each of the three indicators of fiction exposure in explaining variance of the vocabulary sum score (see Table 3 and Fig. 2). The indicators of print exposure were *Z*-standardised, and gender was contrast-coded (−.50 = male, .50 = female). To control for the observed gender differences, the two-way interactions of gender with the self-report scale, book count, and ART-G fiction sub-score were included in the baseline model alongside the ART-G non-fiction sub-score^3^ in order to control for effects of non-fiction exposure. The self-report scale was entered as the predictor in the second model, book count was added in the third model, and the fourth model finally included all three predictors. Multicollinearity was acceptable (all variance inflation factors [VIFs] < 3). *R*^2^ increased significantly in each model. The self-report scale was a significant predictor in the second model but lost its significance when book count was added in the third model. Book count, in turn, predicted vocabulary significantly in the third model but lost its significance when the ART-G fiction sub-score was added in the fourth model. Thus, when all variables—including the ART-G non-fiction sub-score—were entered, the ART-G fiction sub-score remained the only significant predictor of vocabulary (*p* < .001).

**Table 3:** Summary of stepwise multiple regression for the vocabulary sum score (*N* = 293)

Variable	Baseline Model^†^	Model 2			Model 3			Model 4		
		*B*	*SE B*	β	*B*	*SE B*	β	*B*	*SE B*	β
ART-G fiction sub-score	-	-	-	-	-	-	-	3.294	0.660	.437***
Book count	-	-	-	-	1.036	0.514	.137*	0.473	0.506	.062
Self-report scale on frequency of reading fiction	-	1.307	0.433	.172**	0.755	0.510	.100	0.012	0.512	.002
*R*^*2*^	.379	.412			.426			.497		
*F* for change in *R*^*2*^	12.057***	9.094**			4.070*			24.896***		

ART-G = Author Recognition Test–Genres; *** *p* < .001; ** *p* < .01; * *p* < 05; ^†^the baseline model included the intercept, the ART-G non-fiction sub-score, and the following two-way interactions: gender * self-report scale on frequency of reading fiction, gender * book count, gender * ART-G fiction sub-score

## Discussion

There has been a recent increase of research interest in the potential benefits of reading fiction. According to contemporary models (Consoli, 2018; Mar, 2018), investigating the effects of lifetime exposure to written fiction in older adults seems particularly promising. Such a research agenda requires validated indicators of lifetime exposure to print fiction. The present study is the first to look at the three main types of indicators, namely self-report, author recognition test, and book counting, when applied to reading fiction rather than reading in general in a sample of older adults. We investigated convergent and divergent construct validity of the self-report scale and book counting through bivariate correlations with fiction author recognition on the one hand and non-fiction author recognition on the other, and criterion-related validity via associations of each indicator of fiction exposure with vocabulary test scores.

Our first research question addressed whether the self-report scale and book counting are more positively associated with fiction author recognition than with non-fiction author recognition. Such a pattern would indicate that self-report scales and counting fiction books reflect the same or a similar construct as the ART-G fiction sub-score, so that researchers could confine themselves to applying the most efficient measure without loss of information—in that case, using multiple measures would not provide additional information about participants’ engagement with fiction. The correlations observed were all statistically significant and ranged between *rho =* .206 (self-report scale—ART-G non-fiction sub-score) and *rho =* .688 (ART-G fiction—non-fiction sub-score), so were of small to medium size. Importantly, the self-report scale and book counting correlated consistently more strongly with the ART-G fiction sub-score than with the ART-G non-fiction sub-score. In general, the current interrelations of print exposure indicators are within the range of coefficients identified in our review of the literature, though consistently above the 75th percentile. The finding that the present associations were slightly higher than what has been typically found in earlier studies could trace back either to the age of our sample, which is higher than most previous studies, or to the fact that we examined specifically fiction exposure rather than general print exposure.

Whilst the above-mentioned statistics suggest that the self-report scale and book counting have satisfactory levels of convergent validity (Frey, 2018), the variance shared with the ART-G fiction sub-score ranges from 19% to 26%, leaving between 74% and 81% of variations within each indicator unexplained. This means that the constructs assessed by the three measures do overlap partially but are far from congruent (by congruency we mean a shared variance of 100%, or approaching 100% given the levels of noise typically present in empirical observations, rather than the partial overlap found in the current study). Thus, we cannot say from the present data that the three indicators can be used interchangeably. If researchers would like to get a comprehensive picture of participants’ lifetime exposure to print fiction, they might want to apply all three indicators—that is, a self-report-based measure, an author recognition test, and book counting.

The second research question dealt with the strength of associations between each of the three indicators on the one hand and vocabulary test scores on the other. Since vocabulary is considered a central component of reading comprehension (Perfetti & Stafura, 2014), and good levels of reading comprehension at least partly trace back to frequent reading (Perfetti, 1985; Torppa et al., 2020), we assumed that the relation between the indicators and vocabulary test scores would be informative of the indicators’ criterion-related validity. Analyses revealed that, when gender and non-fiction exposure were controlled for, the self-report scale had the lowest predictive value, as its contribution was no longer significant when book counting was added. Book counting proved to be the indicator with the second-best predictive value, as it outperformed the self-report scale but lost its significance when the ART-G fiction sub-score was included as another predictor. Finally, the ART-G fiction sub-score was found to have the highest criterion-related validity, since it emerged as the only significant predictor in the regression model including all three measures of exposure to print fiction. The model including the ART-G fiction sub-score was also the one with the highest *R*^2^, meaning it was the one explaining the most variance of the vocabulary test score. This emphasises the predictive power of the ART-G fiction sub-score.

Compared with earlier evidence summarised in Table 1, the current correlations between indicators of fiction exposure and vocabulary are similar in size, though above the median. Again, slightly higher coefficients could be related to either the age of our sample or the present focus on fiction exposure. Still, it is reassuring that according to the present findings, applying print exposure measures to index fiction exposure in older adults is not associated with reduced, but rather even better, validity.

Additional results partly confirmed and partly deviated from earlier findings. On the one hand, females scored higher than males on all three indicators of exposure to written fiction. This resonates with the well-established finding that females have a stronger preference for fiction texts than males (e.g. Thums et al., 2021). On the other hand, in the current sample none of the measures of fiction exposure were related to age. This conflicts with results of Grolig et al. (2020), where author recognition test scores increased with rising age. The difference between the findings by Grolig et al. (2020) and the present results is likely to reflect differences in the samples’ age ranges. Participants in Grolig et al. (2020) were between 13 and 77 years old, whereas the current sample included a much smaller age range, from 50 to 79 years. Comparatively lower age variance in the present study might have made the detection of an age-based impact more difficult. Whilst we deliberately focused on an older target group to capture lifetime experience with fiction, research on fiction exposure across the entire lifespan would indeed require samples covering the full scope of literate ages.

Although the research reported here makes novel contributions to the field of fiction research in several respects, a few limitations should be acknowledged. First, the skewed distribution of the book count may raise some concerns about the reliability of book counting. The highest possible score of 160 was reported by a percentage (i.e. 30.4 %) more than twice the size expected under normal distribution (here, the score of 160 would be reported by 13.5% only). On one hand, it is possible that the number of fiction books is not normally distributed in our older adult population (who are likely to have accumulated a larger home library over their lifetime compared to the younger samples tested in most previous studies), so assuming normal distribution was incorrect in the first place. On the other hand, it cannot be ruled out that some participants did not actually count their books until they reached 160 but instead made a rough guess, in fact overestimating the number of their books. Unfortunately, we do not have data, such as response times, which could be used to test this potential explanation. Future investigations could compare self-reported book counts with those recorded by researchers during home visits in order to gauge the reliability of self-reported book counts. Figure 1 also reveals that a considerable percentage of the sample (i.e. 7.2%) reported having zero books in their home, which may be counter-intuitive—everyday experience suggests that people typically own at least a small number of books. This result is possibly associated with the target group of the current investigation. Older adults are likely to change their housing situation to something more age-appropriate; either downsizing or moving to a retirement home means adjusting to less personal space, so that one might have to divest oneself of personal belongings including books. In that case, book counting would not reliably reflect lifetime print exposure in this population. Targeted research is needed to clarify this.

Second, the use of a single-item self-report scale likely limited the reliability of this measure. Also, since Schmidt and Retelsdorf (2016) found the SRHI-R, another self-report indicator of print exposure (see Introduction), to be confounded with reading motivation, the same may have applied to the current study. We opted for a single-item scale for three reasons, namely the lack of validated multi-item self-report questionnaires of fiction exposure, a shortage of resources to pilot a new multi-item instrument, and the previous successful application of a bespoke single-item self-report scale in a study with older adults by Payne et al. (2012) . The first issue turned out to be moot, since Kuijpers et al. (2020) developed the Reading Habits Questionnaire to assess fiction and non-fiction exposure. This questionnaire was published after the planning stage of the current study (end of 2019/beginning of 2020) and we were not aware of it until after data collection was completed.

Another limitation is related to the variable used to assess criterion-related validity, namely vocabulary or word knowledge. First, some researchers assume that good word knowledge is the result of frequent reading (e.g. Perfetti, 1985; Perfetti & Stafura, 2014), whereas others postulate other relationships between reading frequency and word knowledge. For instance, performance in a vocabulary test has been shown to predict reading comprehension (e.g. Laufer & Aviad-Levitzky, 2017; Ouellette, 2006), which suggests that word knowledge is a precursor rather than an outcome of reading behaviour. Hence, it remains disputable whether word knowledge is a suitable criterion-variable for reading exposure. Second, even if one accepts word knowledge as an appropriate criterion of print exposure, it does not provide a criterion of *fiction* exposure in particular. General word knowledge should improve through any kind of reading, but a specific benefit after reading fiction is not currently justifiable. However, at present we simply do not know what indicators are suitable criteria for fiction exposure. This may be related to the fact that empirical fiction research is still in its infancy, even though research activities are increasing. Only when research has identified robust outcomes of reading fiction will we learn what measures to apply as external criteria for fiction exposure.

To conclude, in the present study we found evidence to suggest that self-report measures, book counting, and author recognition tests have good levels of construct validity as indicators of exposure to written fiction. However, the three indicators overlap only partially, so that they cannot be used interchangeably. In order to achieve a comprehensive picture of participants’ fiction exposure, researchers are encouraged to apply all three indicators. Out of the measures under investigation, the ART-G fiction sub-score has proven to have the highest criterion-related validity. It remained the only significant predictor of word knowledge both when the impact of gender and non-fiction reading were controlled for and when all indicators were entered in a regression model. Thus, we recommend that researchers include all three measures of fiction exposure if they have the resources to do so, and that they confine themselves to the ART-G in the case that they can include only a single indicator. For the future, it would be interesting to examine the reliability of book counting more closely, and to validate a multi-item self-report scale. Furthermore, estimating criterion-related validity would benefit if reliable outcomes of fiction reading were identified.

## Data Availability

Data are available at the Open Science Framework, https://osf.io/ytudn/. Materials are available from the authors upon request. The research was not preregistered.

## Footnotes

1 This can suggest disingenuous responding to test items, see Wimmer et al. (2021).

2 *N =* 13 participants did not complete the self-report scale on frequency of fiction reading, so the sample size for this measure was *N =* 293 participants.

3 We also considered including the ART-G total score (fiction + non-fiction sub-score) in the baseline model; however, this led to multicollinearity issues (VIFs > 36 for the ART-G total and fiction sub-scores).

## Code availability

The SPSS syntax for the main analyses is available at the Open Science Framework, https://osf.io/sb7xz/.
